# Honey Thieves: Human–Bear Conflict Patterns and Residents’ Attitudes in Mountains of Southwest Zhejiang, China

**DOI:** 10.3390/ani15070922

**Published:** 2025-03-23

**Authors:** Jiale Cheng, Yu Wang, Zihong Zheng, Jin Li, Shanshan Zhao, Xiao Song, Aichun Xu

**Affiliations:** 1 College of Life Sciences, China Jiliang University, Hangzhou 310018, China; jialec2025@163.com (J.C.); zhaoss9211@126.com (S.Z.); 2 Administration Center of Zhejiang Jiulongshan National Nature Reserve, Lishui 323300, China; 15024607567@163.com (Y.W.); zzh19680803@163.com (Z.Z.); 3 Tangpu Reservoir Operation Management, Shaoxing 312300, China; hjyandlj@163.com

**Keywords:** human–wildlife conflict, spatial and temporal patterns, semi-structured interviews, infrared camera, large carnivores

## Abstract

Human–wildlife conflict represents a critical area of research in biodiversity conservation, as understanding both the nature of these conflicts and the attitudes of local communities is essential for developing effective mitigation strategies. In recent years, conflicts between humans and Asiatic black bears (*Ursus thibetanus*) have become increasingly prevalent. Focusing on Jiulongshan Mountain and its surrounding areas in Zhejiang Province, this study conducted a comprehensive investigation into the status of human–bear conflicts and the attitudes of local residents from 2020 to 2023. This study utilized infrared camera monitoring and semi-structured interviews to gather data on conflicts and community perceptions. The following observations were made: (1) Nine infrared cameras recorded 10 instances of Asiatic black bears damaging beehives. (2) Between 2020 and 2023, a total of 186 conflicts involving black bear damage to beehives were reported, resulting in economic losses exceeding RMB 312,000. (3) The peak periods for human–bear conflicts occurred in April–May and November–December, with the sites of these conflicts influenced by topography and levels of human disturbance. (4) A total of 61.0% reported a negative attitude toward Asiatic black bears, 11.9% held a positive view, and 27.1% were neutral. Beekeepers, in particular, exhibited significantly more negative attitudes compared to other occupational groups. (5) Among beekeepers, 52.6% reported taking measures to mitigate losses, such as relocating their hives, building fences, or installing alarms. This study provides a detailed examination of the current state and spatiotemporal patterns of human–bear conflicts in the Jiulongshan Mountain and its surrounding regions. It also offers valuable insights into local residents’ attitudes toward Asiatic black bears, thereby contributing to the development of informed conservation strategies and human–bear conflict mitigation efforts.

## Simple Summary

Conflicts between humans and Asiatic black bears are a growing concern, threatening both wildlife conservation and local livelihoods. This study examined the patterns of human–bear conflicts and the attitudes of residents in Jiulongshan Mountain, Zhejiang Province, China, using infrared cameras and interviews. We found that conflicts were most frequent in spring and winter, and occurred mainly in areas with low human activity. The conflicts exclusively involved damage to beehives, leading to substantial economic losses for local residents. Most residents, especially beekeepers, held negative views of the bears due to these losses. While some residents took measures like relocating beehives or building fences, these efforts were only moderately effective. Our findings highlight the need for better strategies to reduce conflicts, such as providing protective equipment for beehives and educating communities about bear behavior. This research is important for developing solutions that protect both bears and the livelihoods of local residents, promoting peaceful coexistence between human and wildlife.

## 1. Introduction

Human–wildlife conflict has been a persistent issue for millennia, intensifying with the expansion of human activities and the concurrent loss of wildlife habitats [1,2]. Large carnivores, in particular, pose significant challenges to human communities, contributing to crop destruction [3,4], livestock predation [5,6], property damage [7], and, in some cases, direct attacks on people [8,9]. These conflicts not only threaten the survival of endangered species but also exacerbate public intolerance toward wildlife [10,11]. A prominent example of such conflict is the interaction between humans and the Asiatic black bear (*Ursus thibetanus*), a species that not only poses direct threats to human communities but also faces critical conservation challenges due to its endangered status [12,13].

The Asiatic black bear, a member of the genus Ursus in the family Ursidae, is classified as a Class II protected species in China [14]. It is widely distributed across countries such as Japan [9], India [15], Pakistan [8], and China [16]. However, their populations have steadily declined due to habitat degradation, human disturbance, food scarcity, and poaching [17,18], with approximately 50–75% of their historical range lost [19]. In China, estimates place the population of Asiatic black bears at approximately 12,000–18,000 individuals [20]. In response to these threats, the Chinese government has implemented various measures to protect forests and wildlife [21].

Human–bear conflicts are prevalent across the distribution range of Asiatic black bears, particularly in regions such as Japan [9], Pakistan [8], Bhutan [22], China [23,24], and Nepal [25], where conflicts typically manifest as crop destruction, livestock predation, and occasionally human injuries. However, in Jiulongshan Mountain of Zhejiang Province, these conflicts primarily manifest as beehive destruction, with no documented cases of crop damage, livestock predation, or human injuries. This emerging conservation challenge has received disproportionately limited research attention when compared to other conflict types. The phenomenon is particularly evident in Sichuan and Yunnan Provinces, where 60–75% of households report annual beehive losses due to bear intrusions [26,27,28]. Parallel patterns emerge in Pakistan and Bhutan, where Asiatic black bears selectively target apiaries due to honey’s high caloric value, leading to substantial economic losses and retaliatory killings [29,30]. Existing mitigation strategies, including electric fencing and olfactory deterrents, have demonstrated limited efficacy globally, as they frequently neglect fundamental ecological drivers such as seasonal food scarcity [31,32]. These significant knowledge gaps persist in the current research. First, while extensive studies have been conducted in western Chinese provinces (e.g., Sichuan and Tibet), eastern regions, such as Zhejiang, remain understudied, despite escalating human–bear interactions driven by habitat fragmentation and anthropogenic expansion. Second, although anecdotal reports frequently cite beehive damage, its spatiotemporal patterns and socioeconomic impacts remain unquantified in empirical studies. Third, retaliatory measures, including bear mortality events, persist in certain areas, representing unsustainable conflict-resolution approaches [33]. Addressing these gaps through comprehensive analysis of conflict status, causal mechanisms, and spatiotemporal patterns is critical for developing effective management strategies. Such strategies are essential not only for the long-term viability of Asiatic black bear populations, but also for promoting the sustainable development of local communities [34]. Semi-structured interviews have proven to be an effective tool for gathering insights and elucidating these complex socio-ecological dynamics [35].

Jiulongshan Mountain provides a critical habitat and contains the only stable Asiatic black bear population in Zhejiang Province [36]. Since 2018, researchers have employed infrared camera technology to continuously monitor the activity of Asiatic black bears within the reserve. The infrared camera monitoring data from 2020 identified 2–3 individual Asiatic black bears [36]. The most recent infrared camera monitoring data from 2023 identified six individual Asiatic black bears, indicating a growing population trend (unpublished data). This study represents the first comprehensive investigation of human–Asiatic black bear conflicts in Jiulongshan Mountain and its surrounding areas. It aims to (1) characterize the spatiotemporal patterns of these conflicts, with a focus on beehive destruction as the primary conflict type; (2) quantify the socioeconomic impacts of bear-related damage on local communities; (3) assess residents’ attitudes toward bears, particularly among high-risk groups, such as beekeepers; and (4) evaluate the effectiveness of existing mitigation measures to inform future conflict management strategies. Based on previous studies and preliminary observations, we propose the following hypotheses: conflicts peak in spring and winter; hotspots occur on moderate slopes with low human activity; beekeepers hold more negative attitudes; and mitigation measures show limited effectiveness.

## 2. Material and Methods

### 2.1. Study Area

**Figure 1:**
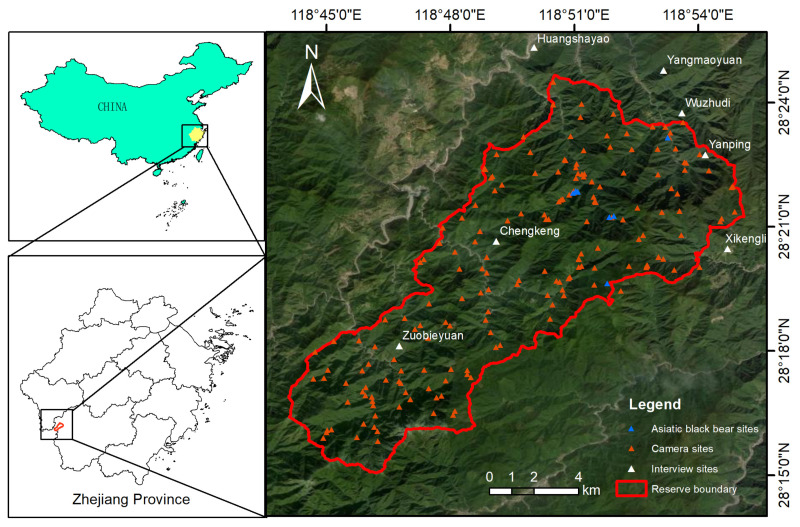
Camera sites, Asiatic black bear sites, and interviewing sites on Jiulongshan Mountain. The study area includes the Zhejiang Jiulongshan National Nature Reserve (red line) and surrounding regions. The red line indicates the boundary of the Zhejiang Jiulongshan National Nature Reserve. Asiatic black bear activity sites represent locations where black bears were recorded by infrared cameras, while camera sites indicate the placement of infrared cameras used for monitoring.

The study area is Jiulongshan Mountain, encompassing the Zhejiang Jiulongshan National Nature Reserve (28°15′~28°24′ N, 118°44′~118°55′ E) and its surrounding regions, established in 2003; this protected area lies within the mid-subtropical monsoon climate zone of the Zhejiang–Fujian hilly region (Figure 1). Jiulongshan Mountain refers to the broader geographical area, while the Zhejiang Jiulongshan National Nature Reserve is a protected area within this region. This area is characterized by distinct seasonal variations and abundant rainfall [37,38]. The annual mean temperature is 15.7 °C, with recorded temperature extremes ranging from a maximum of 38.1 °C to a minimum of −10.6 °C. Annual precipitation averages 1885 mm, distributed across 136 rainy days [39]. The reserve is known for its rich biodiversity, supporting a diverse array of wild flora and fauna. The region’s vegetation predominantly consists of evergreen broadleaf forests, alongside other forest types, contributing to its ecological variety [38]. It also serves as a critical habitat for numerous species of amphibians, birds, and mammals, including the endangered Asiatic black bears [38,39,40]. Beekeeping is a widespread and traditional livelihood activity in the Jiulongshan region [41], with almost 30% of households engaging in beekeeping to some extent. This high proportion reflects the local economic and cultural context, where beekeeping is often practiced alongside other activities, such as farming, forestry, or harvesting wild products (e.g., walnuts). Many households maintain a small number of beehives (e.g., 8–10 hives) as a supplementary income source, rather than relying solely on beekeeping as their primary livelihood.

### 2.2. Data Collection

#### 2.2.1. Infrared Camera Monitoring

Infrared camera technology, particularly infrared-triggered camera trapping, employs infrared sensors to autonomously capture either static images or dynamic footage of wildlife, minimizing the need for human intervention [42]. This method has become a widely adopted tool in wildlife survey research due to its non-invasive nature and effectiveness in monitoring elusive species [43,44,45]. In this study, we used Heron CL-A1 and CL-S1 infrared cameras (Heron, China) for monitoring. A total of 148 independent infrared cameras were deployed across the study area using a 1 km grid system. Cameras were primarily placed along forest paths, which are high-activity zones for Asiatic black bears. The camera locations covered major habitat types, including evergreen broadleaf forests, mixed coniferous and broadleaf forests, deciduous broadleaf forests, coniferous forests, and shrublands, to ensure comprehensive coverage of the study area. An additional 45 infrared cameras were deployed near beehives to specifically record bear-related damage (Figure 1). The monitoring period spanned 12 months, from May 2023 to May 2024. The average number of operational days per camera was 371 (range: minimum of 364 days; maximum of 376 days). The camera data were analyzed to document the frequency and timing of bear activity. The data from the camera traps were combined with beekeeper reports to quantify the spatial and temporal distributions of beehive damage and its socioeconomic impact on local communities. The camera data were used to validate the information obtained from interviews regarding conflicts, as well as to quantify visit frequency and analyze temporal activity patterns using timestamped images.

#### 2.2.2. Semi-Structured Interviews

Semi-structured interviews, valued for their flexibility and capacity to yield detailed, in-depth data, are widely used in studies of human–wildlife interactions [46,47]. This method allows for the exploration of sensitive topics through follow-up questions, while ensuring that all responses are carefully documented [7,48,49]. In this study, interviews were conducted using a structured questionnaire framework, which provided a basis for consistent data collection while accommodating the need for flexibility in addressing specific issues. The sampling design for this study involved the random selection of 7 villages surrounding Jiulongshan Mountain to ensure coverage of the main community types in the study area. Within each village, households were selected through random sampling, and households that had experienced conflicts with Asiatic black bears (e.g., beehive damage and crop loss) were identified through interviews. For households that had experienced conflicts with Asiatic black bears, additional on-site data were gathered regarding the specific sites of these conflicts. In total, 59 households were interviewed in 2023, with data collected on the residents’ demographic information, their perceptions and attitudes toward Asiatic black bears, human–bear conflicts, and the strategies they employed to cope with such conflicts (Table 1). The full questionnaire is provided in Supplementary Materials S1.

### 2.3. Data Analysis

The interview data and camera data were analyzed to identify the temporal and spatial patterns of human–bear conflicts. The annual pattern of human–bear conflicts was analyzed based on the occurrence months by using kernel density estimation (KDE), this analysis was performed using the “ggplot2” package in R Studio 2024.12.1+563 [46,47]. We used the Kernel Density tool in ArcGIS 10.8 to produce a spatial density distribution map of the conflict events. Kernel density analysis was performed using a Gaussian kernel with a bandwidth of 1 km to identify spatial clustering of conflict events. Previous studies have indicated that Asiatic black bears preferentially select habitats characterized by sunny, moderately sheltered ridges or slopes, with a strong affinity for broadleaf forests and mixed coniferous broadleaf forests exhibiting high tree density, substantial canopy cover, and steep slopes [19]. To capture these ecological preferences, six environmental factors were incorporated into the analysis: elevation, aspect, and slope (obtained from the Geospatial Data Cloud, http://www.gscloud.cn, accessed on 10 May 2024), as well as the proximity to water sources, the normalized difference vegetation index (NDVI), and distance to human settlements (derived from the National Geomatics Center of China, http://www.ngcc.cn, accessed on 6 May 2024). These factors were selected for principal component analysis (PCA) to determine the key variables influencing the distribution of human–bear conflicts, a loading < 0.3 is generally considered non-significant [50]. PCA was conducted using SPSS 27.0.

To assess local residents’ attitudes toward Asiatic black bears, we adapted Liu et al. (2011) by categorizing attitudes into three groups: positive, neutral, and negative (Table 1) [3]. Respondents were grouped based on age, gender, and occupation (Table 1). Chi-square tests were used to examine differences in attitudes across various demographic groups, while correlation analyses were conducted using SPSS 27.0 to explore the relationships among the attitude indicators.

## 3. Results

### 3.1. Infrared Camera Shots

Infrared camera monitoring, conducted with cameras deployed throughout the reserve, recorded 10 instances of Asiatic black bears damaging beehives across nine distinct sites (Figure 1). These conflicts occurred within an elevation range of 614–1403 m, spanning a vertical gradient of 702 m. A majority of the recorded conflicts were concentrated between 600 and 800 m elevation, although a few conflicts were observed at higher altitudes.

### 3.2. Patterns of Human–Bear Conflicts

This study collected valid interview data from 59 residents, comprising 53 males and 6 females, with an average age of 53 years. All participants identified as Han Chinese. Regarding their educational background, 10 residents had no formal education, 24 had completed primary school, 19 had completed junior high school, 4 had completed high school, and 2 had attained a university degree or higher. In terms of occupation, 19 participants were beekeepers who primarily relied on beekeeping for their income, occasionally supplementing it with temporary jobs. The remaining residents were primarily engaged in temporary work, with four also serving as forest guards and two operating small businesses.

#### 3.2.1. Types of Conflicts and Economic Losses

A total of 186 human–bear conflicts were recorded in the study area during the study period (2020–2023), all of which involved damage to beehives (Table 2). No conflicts of crop destruction, livestock predation, or human injury were reported. Based on the loss data provided by the residents and estimates of market prices, the total economic losses during this period were approximately RMB 312,000 (Table 2).

#### 3.2.2. Temporal Pattern of Conflicts

**Figure 2:**
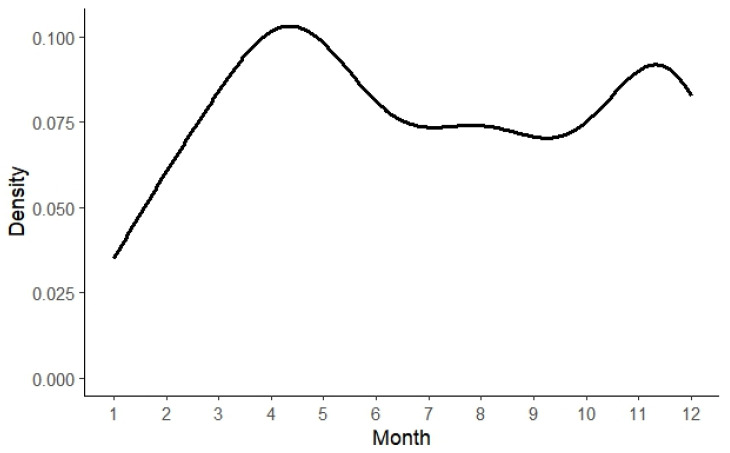
Kernel density curve showing the temporal distribution of human–bear conflicts in Jiulongshan Mountain (total of 196 conflict events). The curve illustrates the frequency of conflicts over time, with peaks in April–May and November–December.

Between 2020 and 2024, a total of 196 conflicts involving Asiatic black bears damaging beehives were recorded, comprising 10 instances captured by infrared cameras and 186 reported through interviews. Due to the extended time lapse between the conflicts and the interviews, the exact month of occurrence was unknown for 29 human–bear conflicts. Statistical analysis (Figure 2) revealed a distinct seasonal pattern in black bear damage to beehives, with conflicts primarily concentrated in the spring months of April and May, as well as the winter months of November and December, resulting in a bimodal distribution.

#### 3.2.3. Spatial Pattern of Conflicts

**Figure 3:**
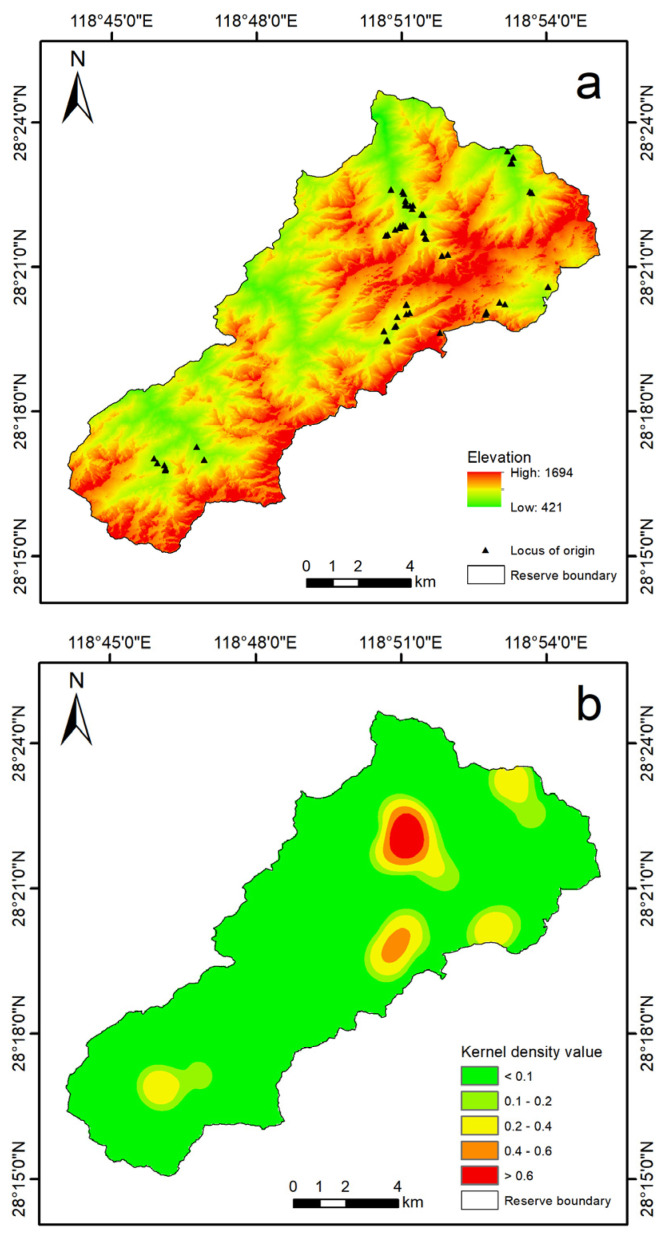
Spatial distribution and kernel density analysis of human–bear conflicts in Jiulongshan Mountain. The study area encompasses the entire Jiulongshan region, but conflicts and black bear activity occurred exclusively within the Zhejiang Jiulongshan National Nature Reserve (Reserve boundary). (**a**) Map showing 56 conflict sites recorded between 2020 and 2023, including 3 sites confirmed by infrared camera monitoring. (**b**) Kernel density map highlighting hotspots of conflict events, generated using a Gaussian kernel with a bandwidth of 1 km.

A total of 196 human–bear conflicts were recorded during the study period, comprising 10 instances captured by infrared cameras and 186 reported through interviews. These conflicts were distributed across 56 distinct sites, with 3 sites confirmed by infrared camera monitoring (Figure 3a). Kernel density analysis revealed significant spatial clustering of conflict events, identifying distinct hotspots primarily concentrated in areas with specific topographical features and lower human disturbance (Figure 3b).

The results of the principal component analysis (PCA) revealed that three principal components had eigenvalues greater than 0.900, with a cumulative variance explained of 71.167% (Table 3). The first principal component exhibited a strong correlation with elevation (correlation coefficient: 0.848) and distance to water sources (0.830), reflecting topographical factors (Table 4), suggesting that black bears prefer higher elevations and areas closer to water. The second principal component was closely associated with aspect (0.727) and slope (0.718), representing additional topographical characteristics (Table 4), indicating a preference for sunny, moderately steep slopes. The third principal component showed a strong correlation with distance to human settlements (0.995), highlighting the influence of human disturbance (Table 4) and the bears’ avoidance of areas with high human activity, likely due to the risk of human disturbance and conflict.

### 3.3. Attitudes and Behaviors of Residents

#### 3.3.1. Residents’ Attitudes

Among the 59 residents surveyed, 61.0% expressed a negative attitude toward Asiatic black bears, primarily due to damage to beehives, perceived threats to personal safety, and the bears’ intimidating appearance. In contrast, 11.9% held a positive view, appreciating the bears’ conservation value and aesthetic appeal, while 27.1% were neutral, citing limited knowledge or minimal direct impact on their lives. Significant differences in attitudes were observed across occupational groups (χ^2^ = 27.453, *p* < 0.001), with all beekeepers expressing negative attitudes (Table 5). No significant differences were found for age (χ^2^ = 8.746, *p* = 0.068) or gender (χ^2^ = 1.067, *p* = 0.448) (Table 5).

#### 3.3.2. Residents’ Responses

A total of 19 beekeeping households reported experiencing black bear intrusions. Among these households, 5 reported the conflicts to government authorities; 4 chose to tolerate the situation; and 10 implemented mitigation measures, including relocating beehives, constructing fences, or installing alarms (Table 6). Specifically, beehives were relocated from high-risk areas to locations closer to human settlements or the edges of the reserve. Fences were constructed using wood or metal, and alarms included motion-activated sirens or noise-making devices. However, the effectiveness of these measures varied (the “Validity Assessment” was based on subjective feedback from the residents): relocating beehives and constructing fences were moderately effective in reducing losses, while alarms proved ineffective due to bears’ ability to adapt to repeated stimuli.

## 4. Discussion

### 4.1. Status and Characteristics of Human–Bear Conflicts on Jiulongshan Mountain

The conflict profile surrounding Jiulongshan Mountain exhibits distinct characteristics compared to established patterns in human–bear interaction research. For instance, the primary conflict types of the multifaceted conflicts documented in other Asiatic black bear habitats, such as the Guthichaur rural municipality in Jumla, Nepal, include crop damage (77.03%), livestock depredation, and human injuries [25]. The primary conflict types in communities surrounding the Gaoligongshan National Nature Reserve in Yunnan, China, in descending order of frequency, include crop damage (57.48%), livestock depredation (20.78%), beehive destruction (19.32%), and human injuries (2.42%) [7]. In contrast, in this study, the local conflicts demonstrated remarkable specificity, being exclusively limited to beehive damage, with no reported cases of crop destruction, livestock predation, or human injuries. The types of conflict were relatively uniform, and the intensity of these conflicts appeared to be relatively low. This may be attributed to the small black bear population in the area [36]. However, two emerging trends necessitate proactive management: (1) the growing Asiatic black bear population, which could escalate the frequency and intensity of conflicts; and (2) significant economic losses from beehive damage, totaling RMB 312,000 (RMB 16,400 per household). These findings contrast with patterns observed in India and Pakistan, where diverse conflict types—including crop raiding, livestock predation, and even human injuries—are documented alongside higher conflict intensity, driven by dense bear populations and extensive human encroachment into forest habitats [8,15]. The unique conflict dynamics in Jiulongshan Mountain highlight the need for targeted mitigation strategies to address both current losses and future risks.

Asiatic black bear behavior is influenced by a combination of geographic, environmental, and food-related factors, which give rise to distinct spatiotemporal patterns of conflicts [51]. This study found that human–bear conflicts are most frequent in the spring and winter. In the spring, Asiatic black bears experience increased energy demands due to reproductive activities [52], making them more likely to raid beehives in search of honey to replenish their energy reserves. In winter, the scarcity of natural food sources prompts Asiatic black bears to adjust their foraging strategies, with honey becoming an important and relatively accessible food resource [52,53]. The spatial distribution of human–bear conflicts on Jiulongshan Mountain is consistent with findings from other regions, such as Yunnan and Sichuan Provinces in China, where conflicts are also concentrated in areas with specific topographical features and lower human disturbance [7,51]. In our study, conflicts were primarily clustered in flat areas away from human settlements; the kernel density analysis and PCA results suggest that black bears prefer higher elevations and areas closer to water, which may explain why conflicts are more frequent in these regions. Additionally, the bears’ avoidance of areas with high human activity may lead to conflicts being concentrated in buffer zones between human settlements and bear habitats, likely due to the bears’ preference for accessible foraging sites and avoidance of high human activity. This pattern aligns with observations in Japan and Sumatra, where black bears frequently raid beehives in remote, low-disturbance areas [9,54].

### 4.2. Attitudes and Behaviors of Rural Residents Toward Asiatic Black Bears Around Jiulongshan Mountain

Understanding residents’ attitudes toward wildlife is critical for effective wildlife management and the mitigation of human–bear conflicts [55], as it provides valuable insights into local acceptance of protected species, awareness of conservation efforts, and the level of support for such initiatives [56,57]. In this study, 61.0% of residents expressed negative attitudes toward Asiatic black bears, 11.9% held positive views, and 27.1% were neutral. Among beekeepers, 100% expressed negative attitudes, indicating particularly strong opposition. This strong opposition is likely due to the economic losses caused by human–bear conflicts. Negative sentiments can pose substantial challenges to wildlife conservation [58]. To mitigate the adverse effects of economic losses, measures such as ecological compensation or insurance schemes [59] could prove effective [60,61]. Some residents chose to tolerate the situation, possibly due to a lack of coping strategies or insufficient knowledge about available solutions. Regarding preventive measures, our findings suggest that relocating beehives and constructing fences were moderately effective, but alarms had limited success due to the bears’ ability to adapt [62]. These results are consistent with studies from Nepal and Bhutan, where metal beehive protection boxes and electric fences have proven highly effective in reducing bear damage [25,30]. Both internationally and domestically, several effective strategies have been developed to address human–bear conflicts [59,60]. For instance, the use of small, independent metal boxes to protect beehives has been shown to significantly reduce economic losses caused by bear raids [63,64]. Implementing such measures in Jiulongshan Mountain could significantly mitigate conflicts, especially given the high economic dependence on beekeeping in the region. However, beekeepers in the Jiulongshan Mountain appear to lack access to relevant information and technical support, leading to the underutilization of these proven methods. This gap requires urgent attention. Although focused on Jiulongshan Mountain in Eastern China, this study reveals universal mechanisms underlying Asiatic black bear–beekeeping conflicts: (1) foraging behavior driven by seasonal food scarcity; (2) habitat fragmentation shaping conflict hotspots; and (3) economic loss as a determinant of human attitudes. Consequently, our mitigation strategies (e.g., metal hive protection boxes and topography-based early warning systems) hold relevance for 1.5 million global beekeepers (FAO, 2022).

Based on the findings of this study, we propose the following recommendations for black bear conservation and the mitigation of human–bear conflicts on Jiulongshan Mountain:

1. Limiting residents’ activities in mountainous forests during peak bear activity periods through public announcements and community education.
2. Providing targeted training and equipment support to beekeepers, including subsidies for metal beehive protection boxes and electric fences.
3. Promoting the use of metal beehive protection boxes through demonstration sites and workshops.
4. Developing early warning systems using motion-activated alarms and camera traps.
5. Exploring livelihood diversification strategies, such as eco-tourism and handicraft production, to reduce dependence on forest resources.
6. Establishing ecological compensation mechanisms and insurance schemes to mitigate economic losses caused by bear damage.

## 5. Conclusions

This study examined human–bear conflicts and residents’ attitudes toward Asiatic black bears on Jiulongshan Mountain. Between 2020 and 2023, 186 conflicts of beehive damage were reported, resulting in economic losses exceeding RMB 312,000. Conflicts were most prevalent in the spring (April–May) and winter (November–December), coinciding with the bears’ breeding season and periods of food scarcity. The spatial distribution of these conflicts was influenced by topographical features and human disturbances. The majority of residents expressed negative attitudes toward Asiatic black bears, primarily due to property damage, with beekeepers exhibiting significantly stronger opposition. The mitigation measures employed by residents, including relocating beehives and constructing barriers, were largely ineffective. To address these challenges, this study recommends enhancing public awareness, providing targeted support to beekeepers, and exploring the implementation of ecological compensation mechanisms to reduce dependence on forest resources.

## Figures and Tables

**Table 1:** Contents of the interview.

Contents of the Interview	Variable	Definition
Basic information of the respondent	Age	<35; 35–55; >55
	Gender	Male or female
	Education level	Unschooled, primary, junior secondary school, high school, university, and above
	Length of residence	>5 years; 6–15 years; 16–30 years; 31–50 years; >50 years
Social and economic information	Source of income	Agriculture; beekeeping; tourism; other
	Income status	Low income; middle income; high income
	Economic loss	Specific amount (RMB)
Black bear damage situations	Type of damage	Beehive damage; crop loss; livestock predation; other
	Time of damage	Months and year
	Sites of damage	Agricultural land; beehives; residential areas; other
Residents’ attitudes and responses	Attitude	1 = like, 0 = neutral, −1 = dislike
	Measures taken	No measures; hive relocation; fencing; alarms; other

**Table 2:** Summary of key findings on human–bear conflicts and residents’ attitudes in Jiulongshan Mountain, Zhejiang Province (2020–2023).

Variable	Category/Description	Results/Values	Remarks
Economic impact	Total economic losses (2020–2023)	RMB 312,000	Average loss of RMB 16,400 per affected household
Conflict types	Damage to beehives	186 incidents	No reports of crop damage, livestock predation, or human injuries
Seasonal patterns	Peak conflict periods	April–May and November–December	Conflicts coincide with breeding season and food scarcity
Spatial patterns	Elevation range of conflict sites	614–1403 m	Majority of conflicts concentrated at 600–800 m elevation
Residents’ attitudes	Negative attitudes	0.610	Beekeepers showed significantly more negative attitudes (χ^2^ = 27.453, *p* < 0.001)
	Positive attitudes	0.119	
	Neutral attitudes	0.271	
Mitigation measures	Proportion of beekeepers adopting measures	0.526	Measures included hive relocation, fencing, and alarm installation (wide area siren and beehive alarm)
	Effectiveness of measures	Hive relocation and fencing: moderate effectiveness. Alarms: ineffective.	Bears adapt to alarms over time

**Table 3:** Eigenvalues and contribution ratios of principal components for habitat selection at human–bear conflict sites. The table shows the eigenvalues, contribution ratios, and cumulative contribution ratios for the six environmental factors analyzed in the principal component analysis (PCA).

No.	Eigenvalue	Ratio of Contribution (%)	Accumulative Ratio of Contribution (%)
1	2.071	34.523	34.523
2	1.204	20.061	54.584
3	0.995	16.584	71.167
4	0.781	13.013	84.180
5	0.547	9.119	93.299
6	0.402	6.701	100.000

**Table 4:** Transposed matrix of eigenvectors for habitat selection at human–bear conflict sites. The table presents the eigenvectors for the six environmental factors (elevation, distance to water sources, aspect, slope, NDVI, and distance to human settlements) analyzed in the principal component analysis (PCA).

Variable	First Principal Component	Second Principal Component	Third Principal Component
Elevation	0.848	0.037	−0.045
Distance to water	0.830	0.175	−0.002
Aspect (slope direction)	−0.332	0.727	−0.017
Slope	0.366	0.718	0.060
NDVI	0.323	0.654	−0.108
Distance from settlements	−0.031	−0.035	0.995

**Table 5:** Mean values of residents’ attitudes toward Asiatic black bears for different demographic variables (gender, age, and occupation). Attitudes were scored as 1 (like), 0 (neutral), and −1 (dislike). The table also includes chi-square test results for differences in attitudes across demographic groups.

Variables	Categories	Mean ± SE	χ^2^	*p*
Gender	Male (*n* = 53)	−0.45 ± 0.08	1.067	0.448
	Female (*n* = 6)	−0.83 ± 0.15		
Age	<35 (*n* = 2)	0.50 ± 0.50	8.746	0.068
	35~55 (*n* = 39)	−0.41 ± 0.12		
	>55 (*n* = 18)	−0.78 ± 0.13		
Careers	Beekeeper (*n* = 19)	−1.00 ± 0.00	27.453	< 0.001
	Alternate (*n* = 40)	−0.25 ± 0.12		

**Table 6:** Statistical data of bear protection measures and assessment of effectiveness. The table summarizes the number of households implementing each measure (e.g., hive relocation, fencing, and alarms), the specificities of each measure, and the subjective assessment of their effectiveness by residents.

Bear-Proofing Measures	Number of Users	No Effect	Weak Effect	Strong Effect	Specificities	Validity Assessment *
Relocation of beehives	10	2	6	2	Beehives were moved from high-risk areas to locations closer to human settlements or reserve edges	Moderate effectiveness in reducing losses
Construction of fences	1	0	1	0	Wooden or metal fences were built around beehives, providing some deterrent effect	Moderate effectiveness, but bears occasionally breached fences
Installation of wide-area alarms	1	1	0	0	Motion-activated alarms mimicking dog or boar noises were installed	Ineffective due to bears’ adaptation over time
Installation of alarms on beehives	2	2	0	0	Sirens were attached to beehives to scare bears away	Ineffective after prolonged use

* Validity assessment of bear-proofing measures as assessed by residents themselves.

## Data Availability

The datasets generated during and/or analyzed during the current study are available from the corresponding author upon reasonable request.

## Supplementary Materials

The following supporting information can be downloaded at https://www.mdpi.com/article/10.3390/ani15070922/s1, Questionnaire S1: Human-Bear Relationship Survey Questionnaire.
