# Supplementary material for: Honey Thieves: Human–Bear Conflict Patterns and Residents’ Attitudes in Mountains of Southwest Zhejiang, China
Source: Animals (Basel). 2025 Mar 23;15(7):922. doi: 10.3390/ani15070922 (PMC11987910; doi:10.3390/ani15070922)
Supplement: Supplementary file 1 [file animals-15-00922-s001.zip › animals-3447583-supplementary.pdf]

Human-Bear Relationship Survey Questionnaire

First of all, thank you very much for taking the time to complete this survey amidst your busy work and studies. The content of this questionnaire is solely for academic research purposes, and your responses will be kept confidential. Please feel free to answer based on your true thoughts. Wishing you progress in your work and studies, and a happy life!

Interview Date: _____Year___Month___Day; Recorder: _________Questionnaire Number:____

______County________Township_________Village______(Small Locality)

I. Basic Information

1.1 Ethnicity: ☐ Han ☐ Other______ Gender: ☐ Male ☐ Female Age:____

1.2 Occupation: ☐ Farmer ☐ Merchant ☐ Cadre ☐ Forest Ranger ☐ Other____________

1.3 Number of Family Members:_______

1.4 Education Level: ☐ No Schooling ☐ Primary School ☐ Junior High School ☐ Senior High School ☐ University and Above

1.5 How many years have you lived in this area? ☐ ≤5 years ☐ 6～15 years ☐ 16～30 years ☐ 31～50 years ☐ >50 years

1.6 Average Annual Family Income: ☐ <10,000 RMB ☐ 10,000～30,000 RMB ☐ 40,000～50,000 RMB ☐ >50,000 RMB

1.7 Source of Income: ☐ Business ☐ Farming ☐ Medicinal Herbs ☐ Migrant Work ☐ Beekeeping ☐ Orchard/Economic Forest ☐ Other______

1.8 Do you regularly enter the mountains?

Yes ☐ How many times per month? ☐ 1 time ☐ 2～10 times ☐ 11～20 times ☐ 21～30 times ☐ >30 times

No ☐

II. Knowledge and Attitudes Towards Black Bears

2.1 How do you know about black bears? (Multiple choices allowed)

☐ Seen in the wild ☐ Seen in a zoo ☐ Seen their footprints or feces ☐ Through books, TV ☐ WeChat Official Accounts ☐ News ☐ Heard from others ☐ Don't know □ Other ways:__

2.2 Do you know that black bears are protected animals?

Yes ☐ Level: Level I ☐ Level II ☐

No ☐

2.3 Do you know if there are black bears in the local area?

Yes ☐ Quantity: ☐ Extinct ☐ Few ☐ Many ☐ Don't know

No ☐

Don't know ☐

2.4 Do you like black bears?

☐ Like very much ☐ Like ☐ Indifferent ☐ Dislike ☐ Hate

Reasons for liking: ☐ Cute appearance ☐ Protected animal ☐ Useful to humans ☐ Religious beliefs ☐ Other:

Reasons for indifference: ☐ Don't know much about black bears ☐ No impact on my life ☐ Other:___________

Reasons for dislike: ☐ Scary appearance ☐ Harm to humans and livestock ☐ Damage to houses ☐ Damage to beehives ☐ Damage to crops ☐ Other:______________

2.5 Compared to 10 years ago, do you think the relationship between humans and black bears has become

☐ More harmonious ☐ Unchanged ☐ Less harmonious ☐ Severe conflict ☐ Don't know

Reasons for the relationship becoming less harmonious:

☐ Black bears are no longer afraid of humans Reasons for black bears no longer being afraid of humans:________________________________

☐ Black bears have learned to use human food ☐ Natural food for black bears has decreased

☐ Black bear population has increased

☐ Ecological restoration has expanded black bear activity range ☐ Don't know

☐ Other reasons:___________________

III. Human-Bear Conflict Situations

3.1 Have you experienced or heard of incidents where black bears harmed humans or livestock, damaged houses, beehives, or crops?

Year	Month	Location	Damage: A:Harm to humans or livestock B: Damage to houses C: Damage to beehives D: Damage to crops E:Damage to beehives F Other (specify)	Bear-proofing Measures	Remarks

3.2 Do you think management measures should be taken for black bears? To what extent?

☐ Strong control ☐ Moderate control ☐ No interference ☐ Moderate protection ☐ Strong protection

3.3 How do you think problematic black bears should be handled?

☐ Drive away ☐ Relocate ☐ Kill ☐ Don’t know ☐ Other:_______

3.4 Do you think local areas need some publicity about black bear protection?

□ Need □ Don’t need
